# Electrochemical Sensing of Curcumin: A Review

**DOI:** 10.3390/antiox12122029

**Published:** 2023-11-22

**Authors:** Ana-Maria Chiorcea-Paquim

**Affiliations:** 1 Instituto Pedro Nunes (IPN), 3030-199 Coimbra, Portugal; anachior@ipn.pt; 2 University of Coimbra, Centre for Mechanical Engineering, Materials and Processes (CEMMPRE), Advanced Production and Intelligent Systems (ARISE), Department of Chemistry, 3004-535 Coimbra, Portugal

**Keywords:** curcumin, polyphenol, phenolic compound, electrochemistry, electrochemical sensing, antioxidant

## Abstract

Curcumin is a natural polyphenol derived from turmeric (*Curcuma longa*) root that has been used for centuries as a spice, coloring agent, and medicine. Curcumin presents anti-inflammatory, antioxidant, anticarcinogenic, antimicrobial, antiviral, antimalarial, hepatoprotective, thrombosuppressive, cardiovascular, hypoglycemic, antiarthritic, and anti-neurodegenerative properties. It scavenges different forms of free radicals and acts on transcription factors, growth factors and their receptors, cytokines, enzymes, and genes, regulating cell proliferation and apoptosis. Curcumin is electroactive, and a relationship between its electron transfer properties and radical-scavenging activity has been highlighted. The objective of this review is to provide a comprehensive overview of the curcumin electron transfer reactions, with emphasis on the controversial aspects related to its oxidation mechanism. The final sections will focus on the electroanalysis of curcumin in natural products, highlighting the most important sensing strategies, based on functional electrodes and nanostructured materials, essential for the development of more efficient in vitro methods of detection and quantification of curcumin in food samples, supplements, and nutripharmaceuticals.

## 1. Introduction

**Figure 1:**
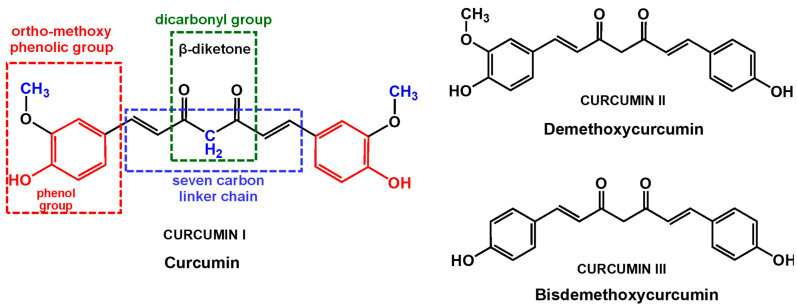
Chemical structures of curcumin (curcumin I), demethoxycurcumin (curcumin II), and bisdemethoxycurcumin (curcumin III).

Curcuminoids are natural phenolic compounds derived from turmeric (*Curcuma longa*) root and other *Curcuma* spp., which are members of the ginger family (*Zingiberaceae*). The main curcuminoid found in turmeric is curcumin (IUPAC name (1E,6E)-1,7-bis(4-hydroxy-3-methoxyphenyl)hepta-1,6-diene-3,5-dione, also known as diferuloylmethane, and curcumin I, Figure 1). Other curcuminoids, such as demethoxycurcumin (curcumin II, Figure 1) and bisdemethoxycurcumin (curcumin III, Figure 1), are also included in the root. Commercial curcumin products usually contain curcumin (approximately 77%), demethoxycurcumin (approximately 17%), and bisdemethoxycurcumin (approximately 3%), indicating that mainly curcumin is consumed from the products [1].

Curcumin has been used for centuries as a spice, coloring agent, and medicinal herb in East Asia. As a spice, it gives the curry its distinctive yellow color and flavor. As a therapeutic agent, curcumin exhibits activity against various diseases, including cancer, cardiovascular diseases, autoimmune disorders, and neurological diseases, such as Alzheimer’s disease, Parkinson’s, and multiple sclerosis. Curcumin possesses numerous pharmacological activities, including anti-inflammatory, antioxidant, anti-carcinogenic, antimicrobial, antiviral, antimalarial, hepatoprotective, thrombosuppressive, cardiovascular (i.e., protection against myocardial infarction), hypoglycemic, antiarthritic (i.e., protection against rheumatoid arthritis), and anti-neurodegenerative (i.e., protection against neurodegenerative diseases).

Curcumin’s pleiotropic activities are generally attributed to its unique chemical structure. Curcumin is a polyphenol from the diarylheptanoid group, that consists of a diferuloylmethane containing two o-methoxy phenolic groups linked to an α,β-unsaturated β-diketone (heptadiene-dione) moiety, Figure 1. The two o-methoxy phenolic groups are important for curcumin anti-inflammatory and antimalarial activities [2,3]. The dicarbonyl group with a reactive central methylene is highly susceptible to nucleophilic attack. Its antioxidant activity is mainly determined by the o-methoxy phenolic and methylenic hydrogen groups. Moreover, the β-diketone group can chelate ions of transition metals, thereby exhibiting enzyme-mimetic antioxidant activities.

Curcumin can act on a variety of molecular targets, such as transcription factors, growth factors and their receptors, cytokines, enzymes, and genes, regulating cell proliferation and apoptosis [1]. It has been shown to improve several systemic markers related to oxidative stress: the plasma activities of superoxide dismutase (SOD) and catalase, and serum concentrations of glutathione peroxidase (GSH) and lipid peroxides [4].

Oxidative stress is directly associated with the majority of chronic diseases, and its pathological processes are closely related to those of inflammation. Therefore, the antioxidant and anti-inflammatory properties of curcumin are considered to be the main mechanisms that explain curcumin’s pharmaceutical benefits. The phenolic and the methoxy groups on the benzene rings and the β-diketone group are the most important structural features that contribute to its antioxidant properties.

An antioxidant is a compound capable of retarding, delaying, or inhibiting the oxidation of a substrate. Depending on their mechanism of action, antioxidants can be divided into primary antioxidants, which act as free radical scavengers, secondary antioxidants, which act by retarding initiation of the chain reaction, and tertiary antioxidants, which are involved in the repair of damaged biomolecules. Curcumin not only scavenges different forms of free radicals, such as reactive oxygen species (ROS) and reactive nitrogen species (RNS), but can also modulate the activity of GSH, catalase, and SOD enzymes active in the neutralization of free radicals, and of ROS-generating enzymes such as lipoxygenase/cyclooxygenase and xanthine hydrogenase/oxidase. In addition, curcumin is a lipophilic compound, which makes it an efficient scavenger of peroxyl radicals, and is considered a chain-breaking antioxidant.

Curcumin content is one of the most important factors in the quality control of commercial turmeric products. In commercially available turmeric products, curcumin varies between 0.5% and 5.7%, depending on growing conditions, harvesting process, and extracting methods. Furthermore, due to the very high cost of pure turmeric, turmeric powder is often adulterated with different chemical compounds, such as lead chromate and metanil yellow, in order to mimic the appearance of curcumin. Various techniques for the qualitative and quantitative analysis of curcumin have been employed, including spectrometric methods [5] and high-performance liquid chromatography (HPLC) [6], but they require expensive instrumentation, difficult sample preparation, and highly trained users. Due to their simplicity, speed, sensitivity, low cost, miniaturization, and portability, the application of electrochemical sensors in routine quality control of natural products and foods, where the curcumin content and possible adulteration and contamination need to be quantified in vitro, is of maximum importance.

Electrochemical methods have received increased attention in studies concerning the redox properties of different classes of phenolic compounds, being able to elucidate their electron transfer reactions, redox potentials, and total antioxidant capacity, and to achieve their electroanalytical determination in highly complex matrixes, such as fruits, vegetables, beverages, food supplements, pharmaceutical drugs, and biological fluids [7,8,9,10]. Understanding the redox behavior of curcumin is essential for understanding its antioxidant activity and can contribute to the development of more efficient in vitro methods of detection and quantification of curcumin in food samples, supplements, and nutripharmaceuticals. In this context, the objective of this review is to provide a comprehensive overview of the fundamental principles concerning curcumin electrochemical behavior, with emphasis on the controversial aspects related to its oxidation mechanism. Moreover, the second part of the review concerns the electroanalysis of curcumin in natural products, highlighting the most important sensing strategies, based on functional electrodes and nanostructured materials.

## 2. Electrochemistry of Curcumin

Curcumin is a phenolic compound that presents three functional groups that can contribute to its biological activity, namely two aromatic o-methoxy phenolic groups, one α,β-unsaturated β-diketone group, and a seven-carbon linker. Its redox behavior is very complex and has led to the proposal of divergent mechanisms [11].

**Figure 2:**
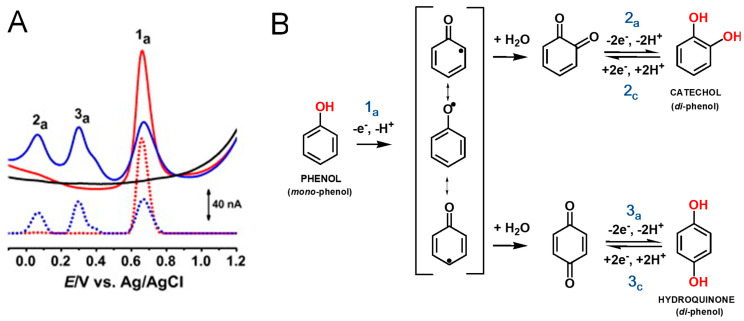
(**A**) Differential pulse voltammograms (DPVs) at GCE in pH 7.0 (▬) supporting electrolytes and 25 μM phenol: (▬) first and (▬) second scans; baseline-corrected DPVs, (•••) first and (•••) second scans; and (**B**) phenol oxidation mechanism. Adapted from [12] with permission.

In general, the redox behavior and antioxidant activity of phenolic compounds are directly related to their phenolic content. Phenol is the aromatic compound that presents one hydroxy (–OH) group linked directly to a benzene ring (Figure 2B).

Phenol undergoes an irreversible, pH-dependent oxidation at the glassy carbon electrode (GCE) that occurs in one step, at the –OH group, with the transfer of one electron and one proton, corresponding to the anodic peak 1a, at *E*_p_ = +0.65 V at pH = 7.0, vs. Ag/AgCl (3 M KCl) (Figure 2A) [12,13,14]. Phenol oxidation leads to the formation of a thermodynamically unstable phenoxy radical that coexists in three resonant forms (at the *ortho* and *para* positions with higher spin density, and at the *meta* position with lower spin density), being immediately stabilized by hydrolysis, resulting in the formation of two electroactive oxidation products, *ortho*-quinone and *para*-quinone (Figure 2B). The *ortho*-quinone and *para*-quinone are further reversibly reduced, the *ortho*-quinone to catechol, peak 3c, and the *para*-quinone to hydroquinone, peak 2c (Figure 2B), in two parallel pH-dependent processes occurring with the transfer of two electrons and two protons each, the reversible peak potentials corresponding to the catechol and hydroquinone electron transfer mechanisms.

Although quite soluble in organic solvents such as dimethyl sulfoxide (DMSO), ethanol, methanol, or acetone, curcumin is poorly soluble in aqueous solvents. Therefore, the first studies concerning curcumin electrochemistry were performed in aprotic solvents. The cyclic voltammetry (CV) of curcumin at GCE in methanol [15] showed the formation of two close oxidation peaks, peak 1a, at *E*_p_ = +0.84 V, and peak 2a, at *E*_p_ = +1.0 V, vs. SCE. The CVs of the curcumin synthetic derivative dimethoxy curcumin that present both –OH groups blocked by CH_3_ groups showed the occurrence of only one oxidation peak, at *E*_p_ = +1.0 V, vs. SCE. The proposed two sites of electrochemical reaction were the phenolic –OH groups and the central CH_2_ group of the β-diketone structure (Figure 1). Comparison with pulse radiolysis studies and DFT calculations suggested that, although the energetics to remove hydrogen from both phenolic –OH and the CH_2_ group of the β-diketone are very close, the phenolic –OH is the most important for curcumin antioxidant activity, being correlated with the oxidation peak 1a. Thus, it was suggested that curcumin undergoes a one-electron process that leads to the formation of phenoxy radicals [15]. Such phenoxyl radical formation was not possible in dimethoxy curcumin, where the phenolic –OH groups are blocked by methoxylation. Therefore, in dimethoxy curcumin, the only observable reaction was the oxidation of the central CH_2_ group, which may also subsequently lose a proton.

In a different report, CV and differential pulse voltammetry (DPV) studies concerning the electrochemical behavior of curcumin at a Pt electrode in acetonitrile also showed that curcumin oxidation is an irreversible and diffusion-controlled process that proceeds in two steps [16].

Curcumin electrochemical behavior was also investigated using direct current (DC) polarography and differential pulse polarography (DPP) in ammonium tartrate aqueous solutions, at pH = 8.1. DPP showed two conjugated reduction peaks, at *E*_p_ = −1.12 V and *E*_p_ = −1.28 V, vs. SCE, while DC polarography showed only one wave, at *E*_1/2_ = −1.28 V, vs. SCE [17]. Using an Al^3+^/Pd nanoparticles (NPs)-modified graphite electrode (GE) in phosphate buffer saline solution at pH = 2, the curcumin oxidation showed one anodic peak, at *E*_p_ = +0.56 V, and two cathodic peaks, at *E*_p_ = +0.50 V and *E*_p_ = +0.30 V, vs. Ag/AgCl (3 M KCl) [18].

**Figure 3:**
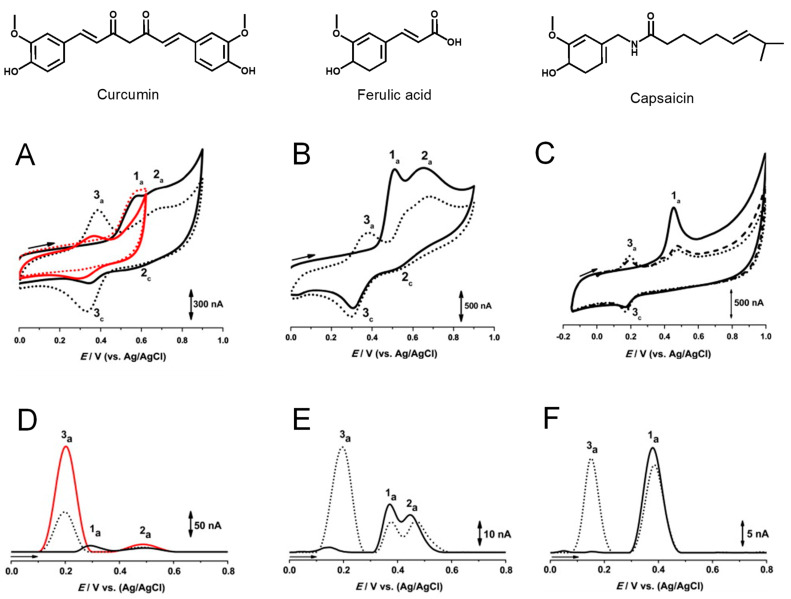
(**A**–**C**) CVs at GCE in a solution of (**A**) 100 μM curcumin at pH = 4.3, (▬) first and (•••) second scans between +0.00 V and +0.90 V, and (▬) first and (•••) tenth scans between +0.00 V and +0.60 V; (**B**) 100 μM ferulic acid at pH = 4.3, (▬) first and (•••) second scans; and (**C**) 10 μM capsaicin at pH = 6.9, (▬) first, (▪▪▪) second, and (•••) third scans, vs. Ag/AgCl (3 M KCl). (**D**–**F**) Baseline-corrected DPVs, (▬) first and (•••) second scans, at GCE in a solution at pH = 6.9 of 10 μM (**D**) curcumin, (**E**) ferulic acid, and (**F**) capsaicin; (**D**,  ▬) adsorbed curcumin oxidation products, first scan in buffer. Adapted from [19] with permission.

The pH dependence of the curcumin redox behavior was investigated at a GCE, using CV, DPV, and square wave voltammetry (SWV), and a redox mechanism for curcumin oxidation was proposed by comparison with the redox behavior of ferulic acid, capsaicin, and dihydrocapsaicin [19]. The oxidation of curcumin (Figure 3A,D) at the GCE revealed to be an irreversible process that in acidic and mild alkaline supporting electrolytes proceeds in two steps [19,20]. The first irreversible oxidation step leads to the formation of a catechol moiety, and the second reversible step occurs for a higher potential. The oxidation of ferulic acid (Figure 3B) was similar to that of curcumin, whereas the oxidation of capsaicin (Figure 3C) and dihydrocapsaicin leads to the formation of only one oxidation product [19].

The adsorption of the curcumin oxidation products at the GCE surface was confirmed when, at the end of several DPVs in the curcumin solution, the GCE was washed with a jet of Milli Q water and transferred to the supporting electrolyte. The DPV obtained in these conditions (Figure 3D (▬)) showed the occurrence of peaks 2a and 3a corresponding to the oxidation of the curcumin oxidation products [19]. Curcumin adsorption onto other carbon-based materials has also been described [21,22,23,24]. Moreover, the physisorption and chemisorptions of curcumin on metal surfaces, which occur through the curcumin’s oxygen atoms and rings π electrons, represent key elements for the efficient use of curcumin as a corrosion inhibitor [25,26].

**Figure 4:**
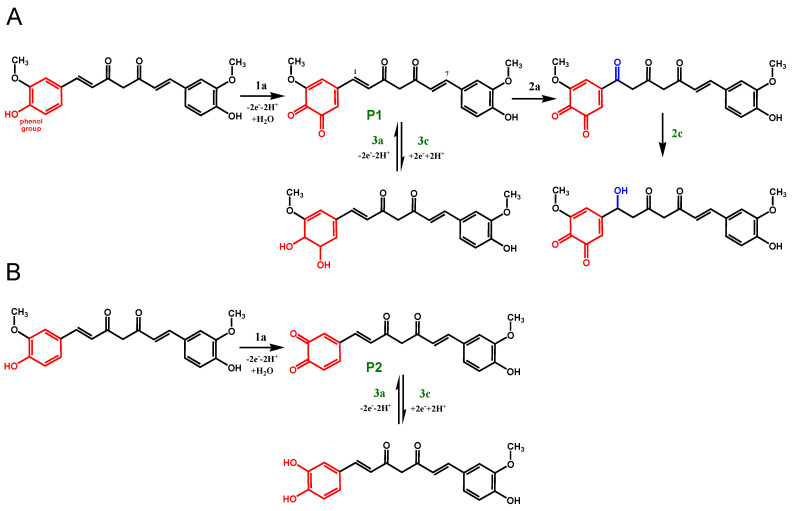
Different oxidation mechanisms of curcumin proposed in the literature. Adapted from (**A**) [19] and (**B**) [24,27] with permission.

A mechanism for curcumin oxidation was proposed, involving the transfer of four electrons and four protons [19] (Figure 4A). Curcumin, ferulic acid, capsaicin, and dihydrocapsaicin all present one –OH group in the benzene ring, which is oxidized at peak 1a, therefore following the oxidation mechanism described for phenol (Figure 2B). It was suggested that at curcumin oxidation peak 1a occurs the formation of the phenoxy radical, which undergoes hydrolysis usually at *ortho* and *para* positions [19]. At the *ortho* position, the phenoxy radical hydrolysis results in the formation of an oxidized product P1 that contains an electrochemically generated *ortho*-quinone moiety and an intact methoxy group. Product P1 is then reduced at peak 3c, and then reversibly oxidized at peak 3a (Figure 3A). The reversible redox reactions, peaks 3a–3c, were always observed for ferulic acid, capsaicin, and dihydrocapsaicin. On the first voltammetric scan, curcumin (Figure 3D) and ferulic acid (Figure 3E) also showed a second oxidation peak 2a, which was not occurring for capsaicin (Figure 3F) and dihydrocapsaicin, indicating that peak 2a is related to the double bond of the hydrocarbon chain of the curcumin and ferulic acid molecules. For a higher potential, it was suggested that curcumin and ferulic acid oxidation peak 2a is due to the oxidation, after hydroxylation at position 1 and/or 7, involving the formation of a product that undergoes reversible redox reactions, peaks 2a–2c (Figure 3A) for curcumin and (Figure 3B) for ferulic acid.

In a different report, the electrochemical oxidation of curcumin, demethoxycurcumin, and bisdemethoxycurcumin (Figure 1) at GCE was investigated by CV and DPV, their products of electrolysis were analyzed by mass spectrometry, and their mechanisms of oxidation were simulated by density functional theory computations [27]. The dependence of current intensities and potentials on pH, concentration, scan rate, and nature of the buffer electrolyte was investigated. The results showed that the oxidation mechanisms of curcumin occur with the transfer of four electrons and four protons, while the oxidation of demethoxycurcumin occurs with the transfer of six electrons and six protons, and of bisdemethoxycurcumin with the transfer of eight electrons and eight protons. Although the formation of the curcumin oxidation product P1 (Figure 4A) was confirmed by electrospray ionization mode mass spectrometry [27], density functional theory computations showed that the formation of curcumin oxidation product P2 (Figure 4B) was preferred over product P1 [27], in agreement with mechanisms proposed for curcumin oxidation at a carbon paste electrode (CPE) [28], carbon black (CB)-modified GCE [29], graphene (Gr)-modified GCE [21], and hanging mercury drop electrode (HMDE) [28]. Curcumin, demethoxycurcumin, and bisdemethoxycurcumin were also investigated at electrochemically reduced graphene oxide (e-rGO)-modified GCE, and a similar oxidation mechanism was proposed [24].

Curcumin voltammetric studies at a functionalized carbon nanotube (f-CNT)-modified GCE in aqueous media showed superior electro-catalytic properties of the f-CNT-modified electrodes in comparison to the GCE in generating the electrochemical response from curcumin [30]. The curcumin oxidation revealed to be a pH-dependent, two-electron–two-proton transfer process.

While many studies considered the curcumin antioxidant activity to be related to curcumin’s ability to donate H atoms from the phenolic groups [15,19,31], pulse radiolysis studies on the formation of curcumin radicals demonstrated that the curcumin antioxidant mechanism involves mostly H-atom abstraction from the central CH_2_ group of the heptadione link, while the H-atom donation from the phenol group only contributes by approximately 15% [32,33]. In addition, subsequent studies showed that the H-atom donation from the phenolic or enolic –OH groups occurs via sequential proton loss electron transfer (SPLET) or hydrogen atom transfer (HAT) [34,35,36].

**Figure 5:**
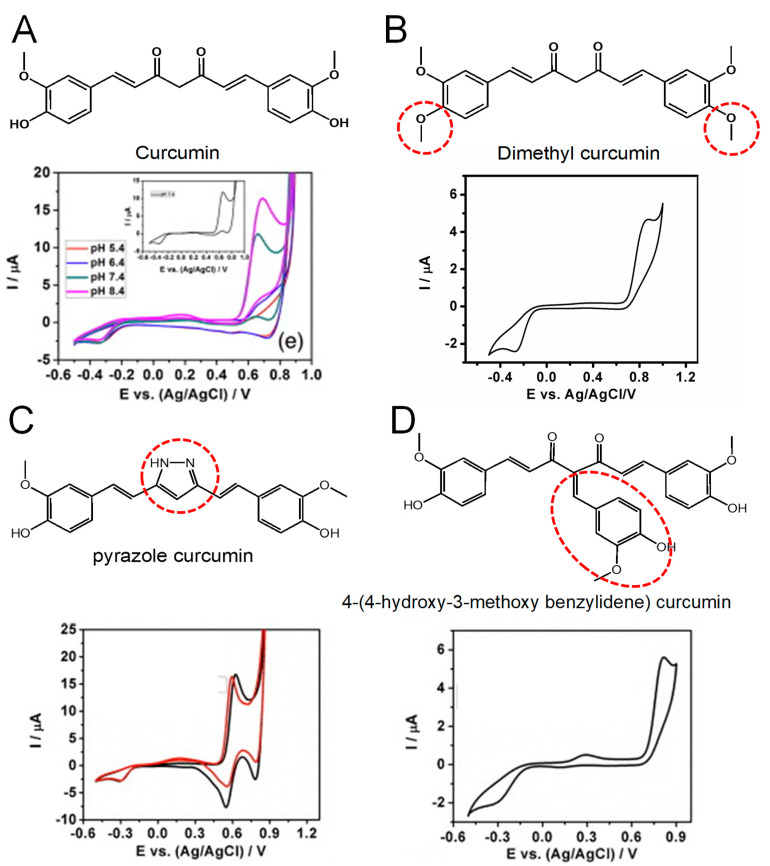
CVs of 8 μM (**A**) curcumin in 10 mM HEPES buffer at various pH values (Inset pH = 7.4, ▬), and (**B**) dimethyl curcumin (▬), (**C**) 4-(4-hydroxy-3-methoxy benzylidene) curcumin (▬), (**D**) pyrazole curcumin (▬), and 3-fluorophenyl pyrazole curcumin (▬) at pH = 7.4; scan rate 20 mV s^−1^. Adapted from [11] with permission.

In order to understand the curcumin electrochemical process and its correlation to its phenolic –OH vs. methylene hydrogen attached to the β-diketone, the curcumin redox behavior at the Au electrode was studied and compared with one of several structurally modified synthetic analogs that abrogate its keto–enol tautomerism or substitute the methylene group at the center of its heptadione moiety implicated in the hydride transfer (Figure 5) [11].

The strong relationship between the curcumin oxidation and its CH_2_ group at the center of the hepatadione link was demonstrated by the redox behavior of dimethyl curcumin that presents both of the phenolic –OH groups blocked [11]. This compound showed only one oxidation peak at higher potentials, at *E*_p_ = +0.87 V in pH = 7.4, vs. Ag/AgCl (3 M KCl) (Figure 5B), this peak being correlated with the central CH_2_ group. Therefore, along with its phenolic –OH groups, curcumin oxidation involves the H atom transfer from the β-alkoxyl radical generated at the center of its heptadione link, which undergoes molecular rearrangement to form the phenoxy radical.

Moreover, the essential role played by the methylene radical at the center of curcumin was confirmed by studying the oxidation of 4-(4-Hydroxy-3-methoxybenzylidene, which lacks the ability of hydride transfer. This compound exhibits lower antioxidant ability and higher redox potential despite possessing an additional ortho-methoxy phenyl group (Figure 5D). Therefore, it can be concluded that these types of compounds that fail to generate β-alkoxyl radicals also compromise the efficient formation of the phenoxy radical [11].

**Figure 6:**
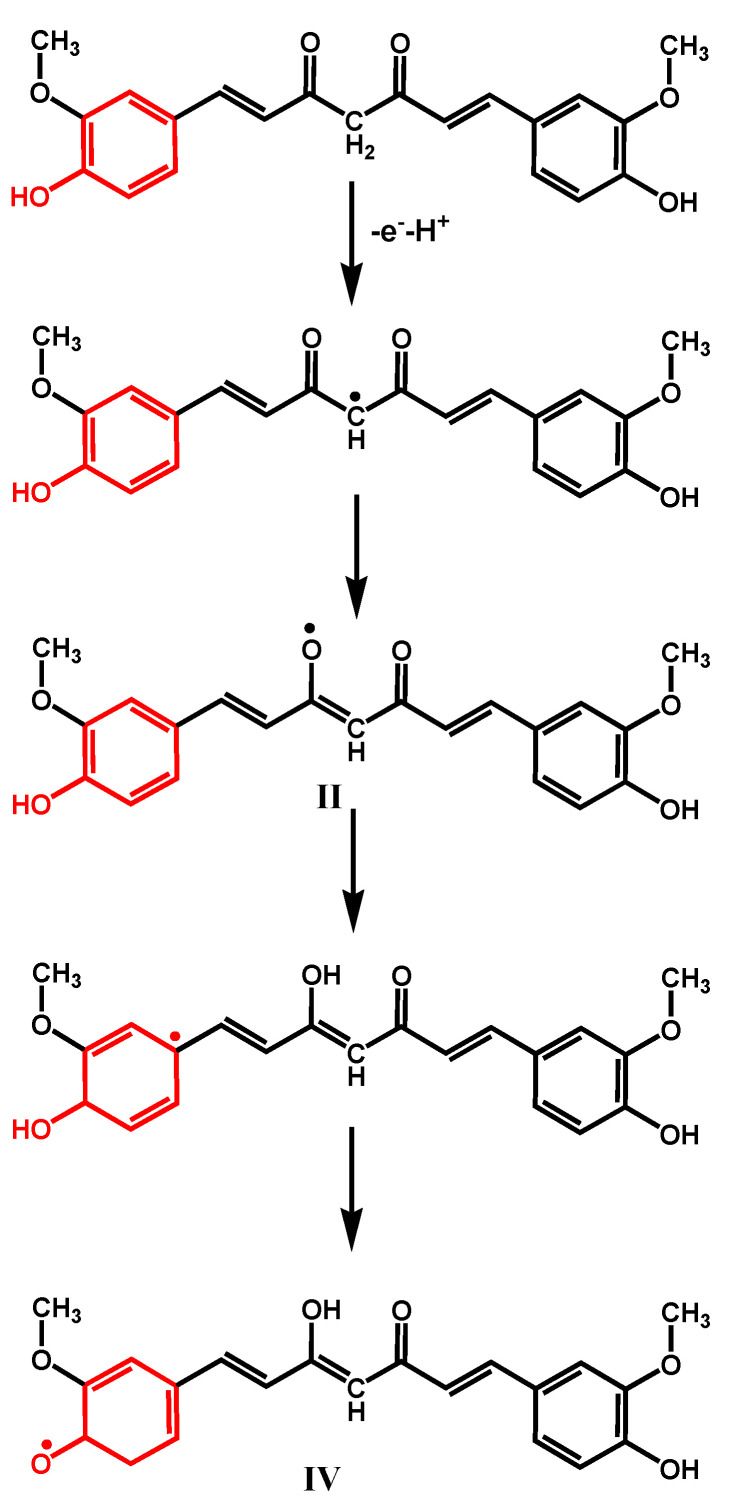
Proposed oxidation mechanism of curcumin. Adapted from [11] with permission.

In addition, the conversion of dicarbonyl moiety of curcumin to an isosteric heterocycle as in pyrazole curcumin, which decreases its rotational freedom, leads to an improvement of its redox properties (lower oxidation potential), as well as its antioxidant activity (Figure 5C). Therefore, it was concluded that the oxidation potential of native curcumin at *E*_p_ = +0.66 V pH = 7.4, vs. Ag/AgCl (3 M KCl) (Figure 5A), is a result of overlapping contributions from the central methylene group and orto-methoxy phenyl group, and a mechanism for the initial formation of the phenoxy radical was proposed (Figure 6).

Corroborating all the results concerning the mechanisms of oxidation of curcumin [11,19,20,24,27], it can be concluded that curcumin oxidation first involves the H atom transfer from the β-alkoxyl radical generated at the center of its heptadione link, which undergoes molecular rearrangement to form the phenoxy radical (Figure 6). This phenoxyl radical further undergoes hydrolysis, most probably at *ortho* position, giving rise to either an oxidized product P1 that contains the methoxy group intact, or an oxidized product P2 (Figure 4). Both P1 and P2 oxidation products contain the electrochemically generated *ortho*-quinone moiety. Density functional theory computations showed that the curcumin oxidation product P2 is preferred over product P1, although the presence of P1 products was confirmed experimentally. Both products P1 and P2 are reduced at peak 3c, and, after, reversibly oxidized at peak 3a. At higher potential, curcumin oxidation peak 2a can be due to the oxidation, after hydroxylation, at position 1 and/or 7, involving the formation of a product that undergoes reversible redox reactions (peaks 2a–2c, Figure 4).

Curcumin antioxidant activity and free radical scavenging ability were also investigated by CV at GCE, using polyaniline as a source of free radicals [20]. The results showed that curcumin exhibits antioxidant activity for polyaniline based on the open circuit potential measurements at both pH 6.8 and 8.0. Moreover, the scavenging ability of curcumin was attributed to the phenolic –OH group at pH 6.8, and to the synergistic effect of the phenolic –OH group and CH group in β-keto-enol at pH 8.0.

In order to understand the influence of carbon nanomaterial on the electrochemical oxidation of curcumin, different nanostructured materials, such as nanofibers (NFs), graphene oxide (GO), activated carbon (AC), functionalized multiwalled carbon nanotubes (f-MWCNTs), single-walled carbon nanotubes (SWCNTs), pristine multiwalled carbon nanotubes (p-MWCNTs), graphitized mesoporous carbon (GMC), graphite nanopowder (GNP), and CB, were examined for curcumin electrochemical oxidation, and it was demonstrated that high surface area and highly porous carbon materials like CB and GMC present the highest electrochemical response for curcumin [29].

The electrochemical behavior of *N*-methyl- and *N*-benzyl-4-piperidone curcumin analogs, with different substituents at the *para* position in the phenyl rings (–H, –Br, –Cl, –CF_3_, and –OCH_3_), were also studied via DPV and CV. The results showed that these types of compounds also suffer a diffusion-controlled, irreversible, two-electron irreversible oxidation in the potential range from +0.72 to +0.86 V [37].

Curcumin is reported to interact strongly with biomacromolecules, and the non-covalent interactions are known to play a decisive role in its mechanism of action on biological targets [38]. Curcumin and curcuminoids and their metal complexes’ interaction with DNA occur through groove binding or intercalation [39]. The interaction between curcumin and calf thymus double-stranded DNA (dsDNA) has been demonstrated by using voltammetry, with DNA electrochemical biosensors built on a CPE or an HMDE [40]. The interaction between calf thymus dsDNA and chromiumin (VI) in the presence of curcumin was also investigated at CPE [41]. In both reports, at CPE, the interaction was followed via the decrease in the oxidation peak of guanine residues in the dsDNA helix after the interaction. The increased DNA damage by the curcumin–chromium complex was observed in the presence of various concentrations of chromium (VI) ions. The results showed that the curcumin–Cr complex causes damage in DNA through the generation of reactive oxygen species, particularly the hydroxyl radical.

The DNA–curcumin interaction was followed also at a hydroxyapatite (HaP) NPs and ionic liquid (IL)-based pencil graphite electrode (PGE) [42]. In a different report, the enhanced electrochemical response from the f-CNT-modified GCE was used to evaluate the chemical and biochemical behavior of curcumin in the presence of transition metal ions copper (II) and dsDNA [30].

## 3. Electroanalytical Determination of Curcumin

Electroanalytical methods of detection have been widely used for the direct, sensitive, and selective determination of curcumin. All electrochemical sensors for curcumin reported in the literature were voltammetric, measuring the current resultant from the curcumin oxidation/reduction processes that take place at the surface of the working electrode. The vast majority of these studies were based on the detection of the curcumin oxidation peak 1a, but very diverse experimental conditions (pH, reference electrode, curcumin solubilization conditions, electrolytes, electrode surface modifications, etc.) have been employed. The most commonly used working electrodes have been the GCE and CPE. However, the poor response of bare electrodes in curcumin direct analysis has led to the use of modified electrodes. Numerous sensing strategies based on functional electrodes and nanostructured materials, such as polymer films, metal and carbon NPs, NFs, nanowires (NWs), quantum dots (QDs), carbon nanotubes (CNTs), Gr, GO, reduced graphene oxide (rGO), metal–organic frameworks (MOFs), and molecularly imprinted polymers (MIPs), have been reported [43,44].

Many research groups have developed innovative electrochemical sensors for the rapid and sensitive detection of curcumin in various complex matrices, ranging from turmeric powder, commercial mixes of seasoning spices, capsules, and liquid supplements, to human plasma, blood serum, and urine. A summary of electrochemical sensors for curcumin quantitative analysis in natural and biological matrixes is presented in Table 1.

### 3.1. Unmodified Electrodes

Based on the curcumin electrochemical reduction peak at HMDE, a simple and rapid differential pulse adsorptive stripping voltammetry (DPASV) determination of curcumin was achieved, in the linear range from 4.9 × 10^−5^ to 2.8 × 10^−5^ M [28]. Later on, the DPASV method, at HMDE in pH = pH 9.5, was used for the determination of curcumin in spiked human serum and turmeric, with a LOD of 1.5 × 10^–9^ M in the linear range from 5.0 × 10^–9^ to 2.8 × 10^–7^ M [63].

Based on the curcumin electrochemical oxidation peak, curcumin determination by DPASV at CPE, in the linear range from 4.9 × 10^−5^ to 2.8 × 10^−5^ M, was also obtained [28], showing a better response when compared with HMDE. A method for curcumin determination at a bare GCE, based on the curcumin oxidation peak at +0.74 V, vs. Ag/AgCl (3 M KCl), in LiClO_4_ solution in ethanol, enabled a LOD of 4.1 × 10^−6^ M in the linear range from 9.9 × 10^−6^ to 1.1 × 10^−4^ M [47]. This method allowed curcumin quantification in a large variety of spices: turmeric M&S, turmeric, freshly milled turmeric root, milled turmeric root after 1 month of storage, seasoning spices for rice and macaroni, and a 15-spice mixture.

A similar LOD of 4.9 × 10^−6^ M in the linear range from 2.2 × 10^−6^ to 4.8 × 10^−4^ M was obtained using an electrochemical method for curcumin and curcuminoid species separation and detection based on their specific chelation with Ni^2+^ ions and adsorptive stripping voltammetry (ASV) at a screen-printed carbon electrode (SPCE) [48]. Recovery of the curcumin from the nickel complex was obtained quantitatively, through acidification of the precipitate. Also based on the high conductivity of NiCl solution and the Ni^2+^ ions’ ability to establish bonds with curcumin, a NiCl_2_-modified GCE sensor was also developed [51], which exhibited a LOD of 1.09 × 10^−7^ M in the linear range from 1 × 10^−6^ to 6.0 × 10^−4^ M, and provided the detection of curcumin in human blood serum

Using an edge plane pyrolytic graphite electrode (EPPGE), direct curcumin DPV determination was achieved with a LOD of 3.0 × 10^−7^ M, in the linear range from 3.2 × 10^−7^ to 2.0 × 10^−6^ M [53]. This was the lowest LOD obtained at unmodified electrodes. Using ultrasonic bath extraction, the curcumin content in turmeric powder was successfully determined.

### 3.2. Polymer-Film-Modified Electrodes

Polymeric film coverage is one of the most attractive ways of sensor surface modification to increase the electrochemical sensors’ sensitivity, requiring minimal use of chemical reagents and volatile organic solvents, ideal for accomplishing the “green chemistry” requirements. For curcumin determination, all electrode modifications were achieved by electropolymerization, which presents the advantage of being very fast and allowing a superior adhesion of the polymer on the electrode surface. However, careful attention has to be paid to the film stability, appropriate layer thickness, and possible contamination.

An electropolymerized titan yellow (PTY)-modified CPE (PTYM-CPE) was used for the quantification of curcumin with a LOD of 1.1 × 10^−6^ M in the linear range from 2.0 × 10^−6^ to 1.0 × 10^−5^ M [45], the method being applied to determine the curcumin content in liquid food supplements with recovery rates of 90.77–105.7%. Additionally, the proposed modified electrode showed easy preparation, elevated sensitivity, stability, and enhanced catalytic activity. In another report, an electropolymerized poly(vanillinco-caffeic acid) (p(Van-CA))-modified Pt electrode was developed that reached a LOD of 5.0 × 10^−6^ M in the linear range from 1.0 × 10^−5^ to 7.0 × 10^−5^ M [49], and showed a curcumin recovery efficiency in turmeric and curry powders of 96–102%. The last report concerned the electropolymerization of acid chrome blue K (ACBK) on a GCE surface. The immobilized poly-ACBK film showed an excellent electrocatalytic response to curcumin when compared with bare GCE. The poly-ACBK/GCE sensor showed a LOD of only 4.1 × 10^−8^ M in the linear range from 1.0 × 10^−7^ to 7.0 × 10^−5^ M [59], with low interference from glucose, lactose, sucrose, NH_4_Cl, NaCl, KNO_3_, CaCl_2_, Cu_S_O_4_, MgCl_2_, Zn(Ac)_2_, citric acid, FeCl_3_, MnCl_2_, and aminoacetic acid, and was successfully applied for the determination of curcumin in human urine samples, with recoveries of 98.7–101%.

### 3.3. Nanostructured-Material-Modified Electrodes

Electrode modifications by carbon-based nanostructured materials emerged as excellent alternatives for bare electrodes, providing chemical inertness, high conductivity, increased surface area, broad working potential, and low background current. Carbon nanomaterials such as CNTs, Gr, GO, rGO, and carbon quantum dots (CQDs) are revolutionary materials that have been widely used in electroanalytical applications. They are super strong and yet highly versatile. However, the main drawbacks are related to cost-effectiveness, the ability to produce them without defects and in uniform sizes, and their potential toxicity, since due to their small size and their strength they can penetrate cellular membranes within the body. Moreover, due to their hydrophobicity, they can easily accumulate and bundle, complicating their usage.

An electrochemical method for the determination of curcumin, based on ASV at a multi-walled carbon nanotube (MWCNT)-modified basal plane pyrolytic graphite electrode (BPPGE), was developed [23]. The MWCNTs-BPPGE showed a LOD of 1.5 × 10^−6^ M in the linear range from 2.0 × 10^−6^ to 1.0 × 10^−4^ M and allowed the determination of curcumin equivalent in turmeric powder samples with recoveries in the range of 92–108%. In a different report, oxidized CNTs that present oxygen-containing functional groups on their surface that increased the surface hydrophobicity were used for the modification of the GCE. The f-CNTs/GCE sensor achieved an excellent LOD of only 6.0 × 10^−8^ M in the linear range from 2.0 × 10^−6^ to 1.4 × 10^−5^ M [30]. Moreover, the interference from several metal ions like Cu^2+^, Zn^2+^, Pb^2+^, Hg^2+^, and Cd^2+^ and organic molecules like dopamine, uric acid, and glucose showed no effect on the sensor selectivity.

A voltammetric sensing platform for curcumin analysis, based on ASV and a MWCTs-modified GCE was also described [65]. The method, although simple, showed a very good LOD of 5.0 × 10^−9^ M in the linear range from 1.1 × 10^−8^ to 5.0 × 10^−6^ M. The methodology was successfully applied to determine the curcumin equivalent in a turmeric powder extract following an ultrasound-assisted extraction. Also based on the MWCNT modification of the GCE, curcumin was detected with an excellent LOD of 5.0 × 10^−9^ M in the linear range from 1.0 × 10^−8^ to 1.0 × 10^−6^ M. It was found that: (i) the influence of Zn^2+^, K^+^, Cu^2+^, Al^3+^, Na^+^, Ag^+^, Mg^2+^, and Ca^2+^, was negligible, (ii) glucose, ascorbic acid, and phenyl alanin amino acid produced a slight decrease in the curcumin oxidation peak due to adsorption and electrode blocking, and (iii) trolox and rutin flavonoids interfered with curcumin determination due to their oxidation peaks overlapping the curcumin oxidation peak. Curcumin quantification in milk solutions with curcumin added as a coloring agent was also shown to be possible [66]. These results were compared with the ones obtained at a dysprosium (Dy) NWs-modified CPE that showed an even lower LOD of only 5.0 × 10^−10^ M for curcumin detection, in the linear range from 2.0 × 10^−9^ to 1.0 × 10^−6^ M [66].

The voltammetric behavior of curcumin and metanil yellow, an azo dye that has been added to turmeric powder to mimic the appearance of curcumin, was studied at a CQD-modified GCE [50]. The metanil yellow oxidation at the CQDs/GCE showed two anodic peaks, at −0.004 V and +0.136 V, and two cathodic peaks, at −0.11 V and −0.05 V, vs. SCE, while curcumin showed two anodic peaks at +0.28 V and +0.55 V and one cathodic peak at +0.25 V. The overlapped voltammograms generally obtained for curcumin and metanil yellow at a bare GCE become well separated at the CQDs/GCE. This strategy allowed curcumin determination with a LOD of 1.0 × 10^−7^ M in the linear range from 4.0 × 10^−7^ to 2.0 × 10^−4^ M, in mixtures of curcumin and metanil yellow, and in turmeric powder samples. Substances like demethoxycurcumin and bisdemethoxycurcumin that undergo oxidation at the same potential as curcumin interfered in the curcumin determination, but not on the metanil yellow determination.

A Gr-modified GCE sensor was used for the detection of curcumin with a LOD of 3.0 × 10^−8^ M in the linear range from 5.0 × 10^−8^ to 3.0 × 10^−6^ M, and good reproducibility [21]. The effect of Gr quantity on the electroanalytical determination was tested. The method was successfully applied to detect curcumin in *Curcuma longa* L. samples with high selectivity (measured against Zn^2+^, K^+^, Ca^2+^, Mg^2+^, Fe^2+^, Na^+^, Cd^2+^, ascorbic acid, glucose, and starch) and accuracy, as well as good recovery.

An rGO-modified CPE showed a LOD of 3.2 × 10^−6^ M in the linear range from 1.0 × 10^−5^ to 6.0 × 10^−3^ M, being used for curcumin determination in human blood serum [46]. e-rGO modification of the GCE showed improved electrocatalytic activity towards curcumin when compared with a bare GCE and GO-modified GCE [24]. The e-rGO/GCE sensor showed a LOD of 1.0 × 10^−7^ M in the linear range from 2.0 × 10^−7^ to 6.0 × 10^−5^ M, also allowing curcumin determination in commercial turmeric capsules, with recoveries of 97–100%.

The excellent ability of Gr-based nanomaterials to improve the sensitivity for curcumin detection was proved by the excellent LODs of 9.0 × 10^−11^ M achieved at a GO-modified GCE (in the linear range from 1.0 × 10^−9^ to 1.0 × 10^−7^ M) and at rGO-modified GCE (in the linear range from 1.0 × 10^−9^ to 1.0 × 10^−8^ M) [71]. The sensors’ selectivity was tested in the presence of Na^+^, K^+^, ascorbic acid, starch, and glucose, with the rGO/GCE showing higher selectivity when compared to GO/GCE.

### 3.4. Nanocomposite-Modified Electrodes

The integration of nanocomposites in the electrochemical sensors substantially improves the electrode-specific surface area, increases the electron transfer, lowers the surface fouling, and decreases the background current. Nanocomposites based on carbon nanostructured materials, metal NPs and NWs, metal oxides, and polymers can considerably increase the sensor response.

With the combination of CNTs with other materials, the performance of the modified electrode can be significantly enhanced. Curcumin was determined electrochemically using a poly(L-arginine) (PAr)-modified CNT paste electrode (CNTPE). Electropolymerization based on L-Arginine amino acid improved the electrocatalytic properties with respect to curcumin detection. The PAr/CNTPE sensor achieved a LOD of 2.2 × 10^−7^ M in the linear range from 2.0 × 10^−7^ M to 5.0 × 10^−6^ M [52], being employed to determine the curcumin content in turmeric powder. The efficacy of the electrode in the concurrent detection of curcumin was examined in the presence of riboflavin and ascorbic acid. A nanocomposite-based sensor with a similar LOD value was developed [18] based on Al^3+^ ions deposited onto a Pd NP-coated GE (Al^3+^/Pd NPs/GE). The sensor showed a LOD of 2.2 × 10^−8^ M in the linear range from 3.0 × 10^−8^ to 6.0 × 10^−7^ M, and enabled curcumin detection in marketed turmeric powder samples.

A sodium dodecyl sulfate (SDS)-modified CPE that enabled the simultaneous determination of curcumin and vitamin B_2_ was also developed [55]. SDS acted as a redox mediator, its functional groups being involved in the electron transfer reactions. The SDS-CPE showed a LOD of 2.7 × 10^−8^ M for curcumin detection in the linear ranges from 2.0 × 10^−7^ to 1.0 × 10^−6^ M and from 1.5 × 10^−6^ to 4.5 × 10^−6^ M, enabling the analyte quantification in natural food supplements. The sensor’s reasonable selectivity was proved in the presence of K^+^, Mg^+^, Na^+^, Zn^+^, ascorbic acid, glucose, starch, tyrosine, and tartrazine. A similar LOD of 2.8 × 10^−8^ M in the linear ranges from 4.0 × 10^−7^ to 6.0 × 10^−6^ and from 6.0 × 10^−6^ to 1.0 × 10^−5^ M was achieved at a poly(glutamine) (PG) film-modified CNTPE prepared using the electropolymerization technique [56]. The sensor showed very good reproducibility and stability, and allowed the detection of curcumin in food supplement samples in mixtures with vanillin. An improved LOD of 8.4 × 10^−8^ M in the linear range from 1.0 × 10^−6^ to 4.8 × 10^−5^ M was achieved at a CNTs-carboxymethylcellulose (CMC) electrode [61], being successfully applied for curcumin quantification in turmeric powder samples.

Metal NP nanocomposites have been important in the development of new electrochemical sensors with improved sensitivity and selectivity because of their catalytic activity, and attractive chemical, optical, and electronic properties. A GE modified by electrodeposition of Pd NPs on polypyroline (poly(Pr)) film was reported [62]. The porous structure of the conducting polymer allowed the dispersion of the Pd NPs into the polymer matrix and generated additional electrocatalytic sites. Using this strategy, a LOD of 1.2 × 10^−9^ M in the linear range from 5.0 × 10^−9^ to 1.0 × 10^−7^ M was achieved.

The rGO’s remarkable properties, such as flexibility, thermal conductivity, charge transport, and capability of coupling electroactive species, in combination with the ability of MWCNTs to impede the agglomeration of rGO nanosheets were used for the development of a sensor consisting of a GCE modified by an azobenzene (Az), rGO, and MWCNT nanocomposite [22]. The Az-rGO@CNTs/GCE was used to detect curcumin with a LOD of 3.0 × 10^−9^ M in the linear ranges from 8.0 × 10^−9^ to 2.0 × 10^−6^ and from 2.0 × 10^−6^ to 1.0 × 10^−5^ M [22], being successfully tested in plasma, urine, and pharmaceutical compounds. Moreover, the sensor showed less than 5% reduction in the curcumin oxidation peak in the presence of interfering compounds.

The correct understanding of interactions at the interface of biological molecules, such as proteins, and nanomaterials is crucial for developing various biocompatible hybrid materials and sensing platforms. Gr-based conductive nanocomposite structures that immobilize proteins can take advantage of the properties of both elements, improving the sensor performance. Using this strategy, two sensors for curcumin detection were developed, which used avidin (Av) conjugated with GO (Av-GO) and rGO (Av-rGO) [64]. The interactive forces between Av and Gr were mainly hydrophobic, along with some van der Waals, electrostatic, and hydrogen bonding interactions, while the structure and function of the Av molecule were largely preserved. The Av-GO-modified GCE showed a LOD of 3.9 × 10^−9^ M in the linear range from 1.0 × 10^−10^ to 1.0 × 10^−7^ M, while the Av-rGO-modified GCE showed a LOD of 4.9 × 10^−9^ M in the linear range from 1.0 × 10^−12^ to 1.0 × 10^−7^ M.

**Figure 7:**
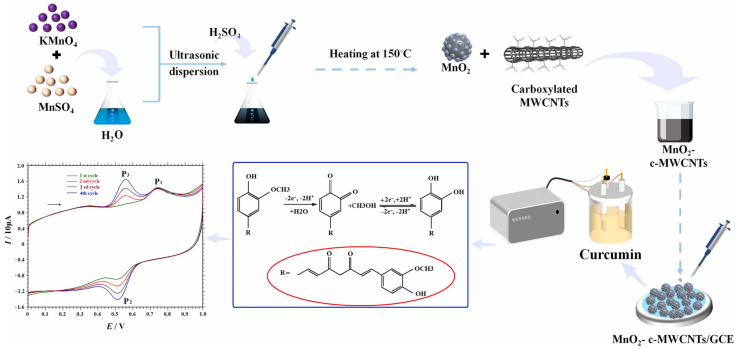
Schematic illustration of the MnO_2_-c-MWCNTs/GCE sensor for the detection of curcumin. Reproduced from [68] with permission.

A sensor consisting of a GCE electrode modified by nanocomposites based on MnO_2_ NPs and carboxylated MWCNTs (MnO_2_-c-MWCNTs/GCE) was recently developed, which showed a LOD of 6.0 × 10^−9^ M in the linear ranges from 1 × 10^−8^ to 1.0 × 10^−6^ and from 1 × 10^−6^ to 8.0 × 10^−5^ M [68] (Figure 7). The sensor was tested in the presence of numerous interfering substances, such as CuCl_2_, ZnSO_4_, NaCl, KNO_3_, MgCl_2_, FeCl_2_, oxalic acid, cysteine, glucose, lactose, sucrose, citric acid, ascorbic acid, vanillin, ferulic acid, L-thiazoline, methionine, phenylalanine, alanine, histidine, glycine, serine, and tyrosine, and no significant effects for curcumin detection were observed. The curcumin content in various food samples (turmeric powder, curry, mustard, instant noodle seasoning, and ginger powder) was also determined.

A further improvement in the LOD of curcumin detection and sensitivity was obtained using a ferrocene (Fc) NFs nanocomposite-modified CPE [69]. The NFs were prepared by electrospinning, based on ZnO NPs and polyvinyl pyrrolidone (PVP), while the Fc-PVP/ZnO NFs-CPE was prepared by mixing Gr powder, Fc, and silicon oil. The sensor exhibited a 2.4 × 10^−10^ M in the linear range from 1.0 × 10^−7^ to 5.0 × 10^−4^ M, and was successfully applied for the measurement of curcumin in urine and turmeric samples. No interference was detected for the presence of Na^+^, Cl^−^, F^−^, S^2−^, CO_3_^2−^, HCO_3_^−^, NO_3_^−^, K^+^, Mg^2+^, Cd^2+^, Ba^2+^, Ni^2+^, Al^3+^, Cu^2+^, Ca^2+^, and Pb^2+^, while carbidopa, ascorbic acid, dopamine, and epinephrine caused interference and more than 5% error in curcumin detection.

In a different report, an electrochemical sensor was fabricated based on a beta-cyclodextrin (β-CD) and rGO nanocomposite immobilized onto a GCE [70]. β-CD’s supramolecular recognition properties, due to its capability to form inclusion complexes with many hydrophobic guest molecules, in combination with the rGO’s high surface area and superconductivity, allowed the construction of a sensor presenting one of the best LODs, of only 3.3 × 10^−10^ M, in the linear range from 5.0 × 10^−8^ to 1.0 × 10^−5^ M. The β-CD/rGO/GCE sensor exhibited good selectivity in the presence of other electroactive species, such as propranolol, clomipramine, and clonazepam.

Up to now, the highest sensitivity and lower LOD for curcumin detection were obtained using a GCE modified by a nanocomposite consisting of Ru@Au NP mixed with L-cysteine-functionalized rGO (NSrGO/Ru@Au NPs) [73]. The sensor showed an excellent LOD of 2.0 × 10^−13^ M in the linear range from 1 × 10^−12^ to 1 × 10^−10^ M and was successfully used for curcumin determination in plasma. To investigate the matrix effect, the developed sensor was applied to a curcumin standard solution, and to a plasma sample containing the same curcumin concentration, and identical voltammograms were obtained, demonstrating the high selectivity of NSrGO/Ru@Au NPs/GCE towards curcumin. The sensor also showed good repeatability, reproducibility, and excellent long-term stability.

### 3.5. Ionic-Liquid-Modified Electrodes

IL-modified electrodes have received considerable attention in electrochemistry due to their good electrical conductivity, and their unique thermophysical and green properties. ILs contain a mixture of positive and negative ions in liquid form, and can be used as binders or modifiers in combination with different nanomaterials, forming hybrid systems with enhanced thermal stability, and improved electrochemical, physical, and chemical properties. A voltammetric sensor for the determination of curcumin in the presence of vitamin B9 was developed, based on a CPE modified by CdO NPs and IL (1,3-dipropylimidazolium bromide as a binder) [60]. The CdO-IL-CPE sensor showed a LOD of 8.0 × 10^−8^ M in the linear range from 2.0 × 10^−7^ to 3.2 × 10^−4^ M, and a good selectivity for curcumin in the presence of a multiple interferents (glucose, methionine, methanol, ethanol monoterpenes, fructose, phenylalanine, L-theronine, lactose, sucrose, alanine, histidine, glycine, Mg^2+^, Na^+^, Ca^2+^, Al^3+^, Cl^−^, F^−^, SO_4_^2−^, K^+^, Br^-^, vanillic acid, ferulic acid, tryptophan, vitamin B2, vitamin B6, cysteine, and serine), being also applied for the determination of curcumin in spices, such as turmeric, seasoning spices for rice and macaroni, freshly milled turmeric root, milled turmeric root stored for 2 months, and an 8-spice mixed seasoning sample.

### 3.6. Molecular-Imprinting-Polymer-Modified Electrodes

MIPs have the ability to specifically recognize template molecules, being considered excellent recognition elements for the construction of electrochemical sensors for curcumin [74]. Generally, they are produced by polymerization in the presence of the target (template) with functional and crosslinking monomers. By removing the template from the built polymer, the template-like cavities become free. These cavities have unique and predetermined selectivity towards their targets, being complementary to their template molecules in shape, size, and chemical functionality. MIP fabrication is also simple and low-cost. However, MIP-based electrochemical biosensors still need to overcome problems related to the formation of heterogeneous binding sites and quite poor synergy with electrochemical detection.

In a first report, an MIP-modified CPE was prepared by mixing the MIP for curcumin target with Gr powder and paraffin oil in specific proportions [54]. The MIP was prepared by thermally induced precipitation polymerization, using curcumin as a template molecule, methacrylic acid as functional monomer, 2,2′-azodiisobutyronitrile as initiator, and ethylene glycol dimethacrylate as cross-linker. The curcumin oxidation peak at the MIP-modified CPE was approximately 4.5 times higher than that at a bare CPE. The sensor achieved a LOD of 1.0 × 10^−8^ M in the linear range from 1.0 × 10^−7^ to 5.0 × 10^−5^ M, and was used to determine the curcumin content in commercial curcuma powder and cookies, with recovery rates of 90.77–105.7%

The development of a low-cost polyacrylic acid (PAA)-based MIP-embedded GE electrochemical sensor for curcumin was also described [57]. This new PAA-MIP/GE sensor showed a LOD of 4.0 × 10^−8^ M in the linear ranges from 1.0 × 10^−6^ to 1.0 × 10^−7^ M and from 1.0 × 10^−7^ to 1.8 × 10^−8^ M. The sensor was tested by studying the effect of various common interfering agents (Ca^2+^, Zn^2+^, Cu^2+^, Fe^3+^, Al^3+^, Cl^−^, Mg^2+^, Na^+^, andrographolide, eugenol, and thymol) on the curcumin peak current, and was successfully applied for the determination of curcumin in raw turmeric, turmeric powder, and capsule samples. Moreover, the sensor presented a high level of reproducibility and stability, the electrodes being successfully stored at room temperature in the air for approximately one month.

**Figure 8:**
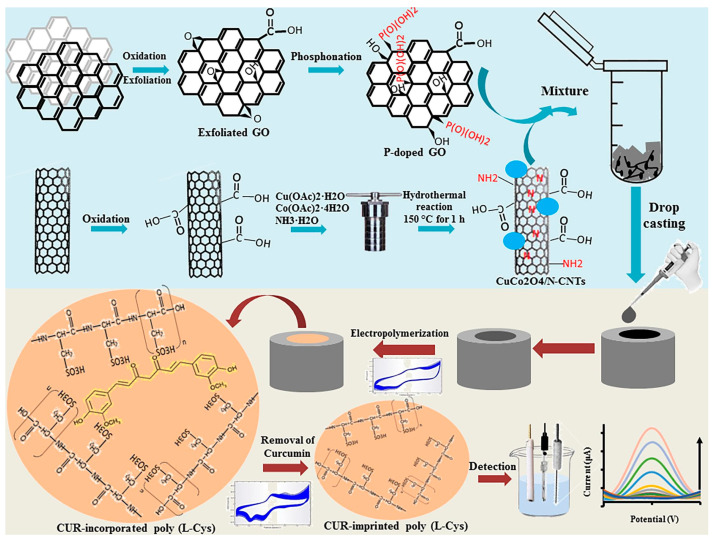
Schematic illustration of the synthesis process of CuCo_2_O_4_/*N*-CNTs and P-GO, and the preparation method of the L-(Cys)-based MIP sensor for curcumin. Reproduced from [58] with permission.

In a different study, an MIP electrochemical sensor for curcumin was fabricated using a highly conductive CuCo_2_O_4_ carbon-based nanocomposite (Figure 8) [58]. In the first step, the GCE electrode was modified by the nanocomposite consisting of CuCo_2_O_4_ bound to *N*-doped CNTs (*N*-CNTs) and P-doped GO (P-GO). In a second step, the CuCo_2_O_4_/*N*-CNTs/P-GO/GCE was further modified by a curcumin-imprinted poly(L-cysteine) (poly(L-Cys)) MIP. L-Cys amino acid was able to electropolymerize and anchor on the surface through the binding of carboxyl, thiol, and amino groups of L-Cys to cobalt and copper. The poly (L-Cys) MIP/CuCo_2_O_4_/*N*-CNTs/P-GO/GCE sensor showed a lower LOD of 3.0 × 10^−8^ M in the linear range from 1.0 × 10^−7^ to 3.0 × 10^−5^ M, and was successfully used for detection of curcumin in biological serum samples with favorable recoveries of 80–110.87%.

Based on a 4-pentenoyl-alanyl-chitosan oligosaccharide (PACO), a functional oligomer of chitosan oligosaccharide, a novel molecular imprinting membrane (MIM) with a strong affinity for curcumin was also developed [67]. The PACO MIM/GCE sensor showed a LOD of 5.0 × 10^−9^ M in the linear range from 1.0 × 10^−8^ to 2.0 × 10^−6^ and was successfully applied to monitor the curcumin content in turmeric extracts with a relative standard deviation of less than 3.3%. Under optimal conditions, the sensor exhibited high sensitivity and good selectivity against some curcumin analogs, such as tetrahydrocurcumin, ferulic acid, β-carotene, and quercetin, and showed good reproducibility, with more than 90% of its original response being retained after storage in a sealed capsule at room temperature for 30 days.

### 3.7. Metal–Organic-Framework-Modified Electrodes

Electrode modifications with MOFs have recently received increased attention since they generally allow superior electrochemical activity, large sizes of pores, and good electrical conductivity. However, in many cases, problems related to MOFs’ tendency to aggregate, which can decrease the electron transfer kinetics due to low conductivity areas, need to be overcome. Although many studies have reported the use of MOFs for the electroanalytical determination of other polyphenols, especially flavonoids, only one study reported porous NPs from a Ce-1,4-benzenedicarboxylic (Ce-BDC) MOF-modified graphite paste electrode (GPE) for the detection of curcumin [72]. The innovative method showed an excellent LOD of 6.0 × 10^−12^ M in the linear ranges from 2.0 × 10^−11^ to 2.0 × 10^−9^ and from 2.0 × 10^−9^ to 9.0 × 10^−9^ M. Moreover, the Ce-BDC MOF-GPE sensor detected both co-mixed curcumin and piperine concentrations without interference, presented an outstanding selectivity in the presence of a large spectrum of interferents (Cl^−^, H_2_PO_4_^−^, CH_3_COO^−^, CO_3_^−2^, SO_4_^−2^, HPO_4_^−2^, PO_4_^−3^, BO_3_^−3^, ascorbic acid, dopamine, glucose, sucrose, cysteine, thiamine, and uric acid), and was able to quantify curcumin in human plasma and urine samples.

## 4. Conclusions

This review presents a comprehensive overview of the curcumin complex redox behavior, with emphasis on its divergent proposed oxidation mechanisms. The development of electrochemical sensors for the detection of curcumin and the reported analytical performance of each sensor were presented.

Electrochemical sensors have been proven to be effective for the detection of curcumin in the presence of interferents and in natural biological matrixes. Due to their high sensitivity, specificity, low cost, easy miniaturization, and use of small amounts of sample, they have become one of the first choices for many researchers and analysts.

A key aspect to consider in the electroanalytical determination of curcumin is the choice of electrode material and sensor architecture. The most commonly used working electrode materials have been glassy carbon and carbon paste. However, to enhance the electron transfer, improve the curcumin-capture effectiveness, and amplify the electrochemical response, various nanostructured materials, including metal and carbon nanoparticles, nanofibers, nanowires, quantum dots, carbon nanotubes, graphene, graphene oxide, reduced graphene oxide, MOFs, and MIPs, have been used. Among them, the most promising results were observed for the electrochemical sensors based on nanocomposites and MOFs. Nanocomposites and MOFs based on carbon nanostructured materials, metal NPs and NWs, metal oxides, and polymers considerably increased the sensitivity, selectivity, accuracy, and repeatability of these sensors. Surprisingly, MIP-based sensors showed only medium sensitivities and limits of detection. Therefore, further studies are required to better take advantage of the MIPs’ cost-effectiveness, excellent thermal and chemical stability, and long shelf life without loss of affinity for the target analyte, while mitigating their drawbacks, by increasing their catalytic capabilities and their binding site homogeneity.

Owing to the complexity of the food and biological systems, developing electrochemical sensor devices for curcumin is challenging. Additional research and analysis should enhance curcumin quantification in natural food matrixes, where other polyphenols, especially flavonoids, are usually present, their anodic peaks overlapping with the curcumin anodic peak, thus complicating its detection. Future research directions should also include sample preparation methods able to minimize the effect of other polyphenolic interferents.

Other challenges in the development of electrochemical detection methods for curcumin are related to the sensors’ short shelf life, and fluctuations in the response depending on pH or temperature, which have to be overcome. Additionally, miniaturization and mass production of compact sensors is an emergent need in the food industry, where the curcumin content and possible adulteration and contamination need to be quantified, without the need to use trained laboratory professionals. Lowering the cost of some of these sensors is also necessary to increase their use in common quality control assays in the field. Despite these disadvantages, electrochemical biosensors for curcumin detection have gained increasingly more attention for the quality control of natural products, foods, and supplements. Further studies could overcome the current disadvantages, lowering the detection limits of the current sensors.

## Figures and Tables

**Table 1:** Electrochemical sensors for curcumin detection, presented in decreasing order of LOD.

Sensor	Method	Linear Range (M)	LOD (M)	Matrix	Ref.
HMDE	DPASV	4.9 × 10^−5^–2.8 × 10^−5^	—	—	[28]
CPE	DPASV	4.9 × 10^−5^–2.8 × 10^−5^	—	—	[28]
PTY-CPE	CV	2.0 × 10^−6^–1.0 × 10^−5^	1.1 × 10^−6^	food supplement (liquid)	[45]
MWCNTs-BPPGE	ASV	2.0 × 10^−6^–1.0 × 10^−4^	1.5 × 10^−6^	turmeric powder	[23]
rGO-CPE	DPV	1.0 × 10^−5^–6.0 × 10^−3^	3.2 × 10^−6^	human blood serum	[46]
GCE	CV	9.9 × 10^−6^–1.1 × 10^−4^	4.1 × 10^−6^	turmeric M&S, turmeric, freshly milled turmeric root, milled turmeric root (1-month storage), seasoning spices for rice and macaroni, 15 mixed spices	[47]
SPCE	ASV	2.2 × 10^−6^–4.8 × 10^−4^	4.9 × 10^−6^	artificial capsaicin (*N*-vanillylnonanamide)	[48]
p(Van-CA)/Pt	DPV	1.0 × 10^−5^–7.0 × 10^−5^	5.0 × 10^−6^	turmeric powder, curry powder	[49]
e-rGO/GCE	DPV	2.0 × 10^−7^–6.0 × 10^−5^	1.0 × 10^−7^	commercial turmeric capsules	[24]
CQDs/GCE	DPV	4.0 × 10^−7^–2.0 × 10^−4^	1.0 × 10^−7^	turmeric powder	[50]
NiCl_2_/GCE	DPV	1.0 × 10^−6^–6.0 × 10^−4^	1.1 × 10^−7^	human blood serum	[51]
PAr-CNTPE	DPV	2.0 × 10^−7^–5.0 × 10^−6^	2.2 × 10^−7^	turmeric powder	[52]
EPPGE	DPV	3.2 × 10^−7^–2.0 × 10^−6^	3.0 × 10^−7^	turmeric	[53]
MIP-CPE	CV	1.0 × 10^−7^–5.0 × 10^−5^	1.0 × 10^−8^	curcuma powder, curcuma cookies	[54]
SDS-CPE	DPV	2.0 × 10^−7^–1.0 × 10^−6^ 1.0 × 10^−6^–4.5 × 10^−6^	2.0 × 10^−8^	food supplement	[55]
PG-CNTPE	DPV	4.0 × 10^−7^–6.0 × 10^−6^ 6.0 × 10^−6^–1.0 × 10^−5^	2.8 × 10^−8^	food supplement	[56]
Al^3+^/Pd NPs/GE	SWV	3.0 × 10^−8^–6.0 × 10^−7^	2.2 × 10^−8^	turmeric powder	[18]
Gr/GCE	LSV	5.0 × 10^−8^–3.0 × 10^−6^	3.0 × 10^−8^	*Curcuma longa L.*	[21]
PAA-MIP/GE	DPV	1.0 × 10^−6^–1.0 × 10^−7^ 1.0 × 10^−7^–1.8 × 10^−8^	4.0 × 10^−8^	raw turmeric, turmeric powder, capsules	[57]
poly(L-Cys) MIP/ CuCo_2_O_4_/*N*-CNTs/P-GO/GCE	DPV	1.0 × 10^−7^–3.0 × 10^−5^	3.0 × 10^−8^	serum sample	[58]
poly-ACBK/GCE	DPV	1.0 × 10^−7^–7.0 × 10^−5^	4.1 × 10^−8^	human urine	[59]
f-CNTs/GCE	SWV	2.0 × 10^−6^–1.4 × 10^−5^	6.0 × 10^−8^	—	[30]
CdO-IL-CPE	SWV	2.0 × 10^−7^–3.2 × 10^−4^	8.0 × 10^−8^	turmeric, seasoning spices for rice and macaroni, freshly milled turmeric root, milled turmeric root (2-month storage), 8 mixed spices	[60]
CNTs-CMC	CV	1.0 × 10^−6^–4.8 × 10^−5^	8.4 × 10^−8^	turmeric powder	[61]
Pd NPs/poly(Pr)/GE	SWV	5.0 × 10^−9^–1.0 × 10^−7^	1.2 × 10^−9^	—	[62]
HMDE	ACSV	5.0 × 10^–9^–2.8 × 10^–7^	1.5 × 10^–9^	human serum, turmeric	[63]
Az-rGO@CNTs/GCE	SWV	8.0 × 10^−9^–2.0 × 10^−6^ 2.0 × 10^−6^–1.0 × 10^−5^	3.0 × 10^−9^	plasma, urine, pharmaceuticals	[22]
Av-GO	CV	1.0 × 10^−10^–1.0 × 10^−7^	3.9 × 10^−9^	—	[64]
Av-rGO	CV	1.0 × 10^−12^–1.0 × 10^−7^	4.9 × 10^−9^	—	[64]
MWCTs/GCE	ASV	1.1 × 10^−8^–5.0 × 10^−6^	5.0 × 10^−9^	turmeric powder	[65]
MWCNT/GCE	FLE-FFTSWV	1.0 × 10^−8^–1.0 × 10^−6^	5.0 × 10^−9^	milk with a curcumin coloring agent	[66]
PACO MIM/GCE	DPV	1.0 × 10^−8^–2.0 × 10^−6^	5.0 × 10^−9^	turmeric extract	[67]
MnO_2_-c-MWCNTs/GCE	SDLSV	1.0 × 10^−8^–1.0 × 10^−6^ 1.0 × 10^−6^–8.0 × 10^−5^	6.0 × 10^−9^	turmeric powder; curry; mustard; instant noodle seasoning; ginger powder	[68]
Fc-PVP/ZnO NFs-CPE	SWV	1.0 × 10^−7^–5.0 × 10^−4^	2.4 × 10^−10^	turmeric, urine	[69]
β-CD/rGO/GCE	DPV	5.0 × 10^−8^–1.0 × 10^−5^	3.3 × 10^−10^	—	[70]
Dy-NW-CPE	FLE-FFTSWV	2.0 × 10^−9^–1.0 × 10^−6^	5.0 × 10^−10^	milk with a curcumin coloring agent	[66]
GO/GCE	CV	1.0 × 10^−9^–1.0 × 10^−7^	9.0 × 10^−11^	—	[71]
rGO/GCE	CV	1.0 × 10^−9^–1.0 × 10^−8^	9.0 × 10^−11^	—	[71]
Ce-BDC MOF-GPE	SWV-ASV	2.0 × 10^−11^–2.0 × 10^−9^ 2.0 × 10^−9^–9.0 × 10^−9^	6.0 × 10^−12^	plasma, urine, co-mixed with piperine	[72]
NSrGO/Ru@Au NPs/GCE	SWV	1.0 × 10^−12^–1.0 × 10^−5^	2.0 × 10^−13^	plasma	[73]

Abbreviations: 4-pentenoyl-alanyl-chitosan oligosaccharide (PACO); acid chrome blue K (ACBK); adsorptive cathodic stripping voltammetry (ACSV); adsorptive stripping voltammetry (ASV); avidin (Av); azobenzene (Az); basal plane pyrolytic graphite electrode (BPPGE); beta-cyclodextrin (β-CD); cadmium oxide (CdO); carbon nanotube paste electrode (CNTPE); carbon paste electrode (CPE); carbon quantum dots (CQDs); carboxylated MWCNTs (c-MWCNTs); carboxymethylcellulose (CMC); Ce-1,4-benzenedicarboxylic (Ce-BDC); differential pulse adsorptive stripping voltammetry (DPASV); dysprosium (Dy); edge plane pyrolytic graphite electrode (EPPGE); electrochemically rGO (e-rGO); flow injection electrochemical–fast Fourier transform SWV (FLE-FFTSWV); functionalized CNTs (f-CNTs); glassy carbon electrode (GCE); graphene (Gr); graphite paste electrode (GPE); hanging mercury drop electrode (HMDE); ionic liquid (IL); L-cysteine functionalized rGO (NSrGO); linear sweep voltammetry (LSV); metal–organic framework (MOF); molecular imprinting membrane (MIM); molecularly imprinted polymer (MIP); multi-walled carbon nanotubes (MWCNTs); nanowire (NW); *N*-doped CNTs (*N*-CNTs); P-doped GO (P-GO); poly(glutamine) (PG); poly(L-arginine) (PAr); poly(L-cysteine) (poly(L-Cys)); poly(vanillinco-caffeic acid) (p(Van-CA)); polyacrylic acid (PAA); polymerized titan yellow (PTY); polypyroline (Poly(Pr)); reduced graphene oxide (rGO); screen-printed carbon electrode (SPCE); second-order derivative LSV (SDLSV); sodium dodecyl sulfate (SDS); square wave voltammetry (SWV).

## Data Availability

No new data were created or analyzed in this study. Data sharing is not applicable to this article.
